# Role of Bovine Serum Albumin Addition in Micellization and Gel Formation of Poloxamer 407

**DOI:** 10.3390/polym15112465

**Published:** 2023-05-26

**Authors:** Namon Hirun, Pakorn Kraisit, Siriwat Soontaranon

**Affiliations:** 1 Thammasat University Research Unit in Smart Materials and Innovative Technology for Pharmaceutical Applications (SMIT-Pharm), Faculty of Pharmacy, Thammasat University, Pathumthani 12120, Thailand; pakorn54@tu.ac.th; 2 Synchrotron Light Research Institute (Public Organization), Nakhon Ratchasima 30000, Thailand; siriwat@slri.or.th

**Keywords:** poloxamer 407, bovine serum albumin, thermoresponsive gelation, micellization

## Abstract

The combination of the thermoresponsive polymer and protein has demonstrated great promise in its applications in drug delivery and tissue engineering fields. This study described the impact of bovine serum albumin (BSA) on the micellization and sol–gel transition behaviors of poloxamer 407 (PX). The micellization of aqueous PX solutions with and without BSA was examined using isothermal titration calorimetry. In the calorimetric titration curves, the pre-micellar region, the transition concentration region, and the post-micellar region were observed. The presence of BSA had no noticeable impact on critical micellization concentration, but the inclusion of BSA caused the pre-micellar region to expand. In addition to studying the self-organization of PX at a particular temperature, the temperature-induced micellization and gelation of PX were also explored using differential scanning calorimetry and rheology. The incorporation of BSA had no discernible effect on critical micellization temperature (CMT), but it did affect gelation temperature (T_gel_) and gel integrity of PX-based systems. The response surface approach illustrated the linear relation between the compositions and the CMT. The major factor affecting the CMT of the mixtures was the concentration of PX. The alteration of the T_gel_ and the gel integrity were discovered to be a consequence of the intricate interaction between PX and BSA. BSA mitigated the inter-micellar entanglements. Hence, the addition of BSA demonstrated a modulating influence on T_gel_ and a softening effect on gel integrity. Understanding the influence of serum albumin on the self-assembly and gelation of PX will enable the creation of thermoresponsive drug delivery and tissue engineering systems with controlled gelation temperatures and gel strength.

## 1. Introduction

Over the past few years, the self-assembly of amphiphilic copolymers has increased attention. It has been considered for various applications, such as templates for nanomaterial synthesis, vehicles for drug encapsulation, and matrices for drug delivery and tissue engineering [1,2,3]. Among the several amphiphilic copolymers, poloxamers are becoming more prevalent in the biochemical and pharmaceutical fields due to their minimal immunological response and low toxicity [3,4,5]. The triblock structure of poloxamers consists of a central hydrophobic poly(propylene oxide) (PPO) block and terminal hydrophilic poly(ethylene oxide) (PEO) blocks. The amphiphilicity of poloxamers depends on the molecular weight and the number and proportion of PEO and PPO blocks [3]. The self-assembly of the unimers in an aqueous environment varies according to polymer concentration and temperature [6]. Moreover, the addition of cosolvent and some additives refines the self-assembly of poloxamers in water [4,5,7]. The worsening interaction between the hydrophobic block of the polymer chain and water with a rise in temperature triggers heat-induced self-assembly in aqueous poloxamer solutions, resulting in micelle formation [5]. The formation of the polymeric micelles and further micelle organization relate to the alteration of physicochemical behaviors such as rheological characteristics, encapsulation performance, and thermogelation ability [5,7]. Since the underlying self-assembly behavior governs the structural features and applications, several efforts have been made to tune, characterize, and comprehend the additive impact on the phase transitions of poloxamers [5].

Poloxamer 407 (PX), also known as pluronic F127, is one particular type of amphiphilic copolymer belonging to a family of poloxamers. The self-organization of PX unimers in aqueous solutions has become an emerging topic with fundamental as well as technological aspects that arise from their capacity to form micellar liquids and physical soft gels [5,8]. At a certain temperature, most of the PX unimers form micelles when the polymer concentration reaches a critical micellization concentration (CMC). The CMC value of PX in water at 30 °C has been reported to be in the range of 0.04 to 0.1 % *w*/*v* [9,10,11]. Apart from the concentration-dependent self-organization behavior, the aqueous solutions of PX become micellar liquids with increasing temperature. By raising the PX concentration above the CMC or by elevating the temperature to a critical micellization temperature (CMT), the process of self-organization can be triggered. Furthermore, PX solutions show thermoresponsive gelation, changing from the low-viscosity micellar liquid to the viscoelastic gel upon heating as a result of the development of an orderly packed inter-micellar structure. At concentrations greater than 15% *w*/*v*, aqueous PX solutions display the thermoresponsive gelation feature [12]. The temperature at which the change from liquid to gel happens is known as a gelation temperature (T_gel_) [13]. Although the thermoresponsive gelation makes aqueous PX solution an appealing thermoresponsive system for drug delivery and tissue engineering, the low T_gel_ of plain PX solution is a drawback and limits its potential performance [5,12,14]. To avoid the usage of new synthetic polymer derivatives with potential toxicities, the search for suitable small-molecular or macromolecular additives is a promising strategy for tailoring the T_gel_ of the aqueous PX solution [5,15]. In addition, understanding the impact of additives on the temperature-dependent characteristics of PX-based solutions is of great importance for the development of the thermoresponsive system [15]. The various types of poloxamers have their own specific molecular weights and proportions of PEO and PPO blocks [16]. The hydrophilic–hydrophobic balance of each poloxamer is associated with the PEO/PPO ratio in the chain structure [17,18,19]. Poloxamer self-assembly in water is more likely to occur when the polymer possesses more hydrophobic PPO [17]. The PEO/PPO ratio in PX is lower than that in poloxamer 188 [20]. At temperatures below 100°C, PX at concentrations greater than 15% can form gel, while poloxamer 188 at concentrations below 40% cannot form gel [21]. In addition, it has been reported that poloxamer mixtures rich in hydrophobic PPO have low T_gel_, whereas solutions rich in hydrophilic PEO have a high T_gel_ [20,22]. The micellization and gelation of PX-based matrices can be altered by combining PX with another type of poloxamer containing different lengths of the hydrophobic and/or hydrophilic blocks. The thermal and rheological properties of systems containing PX and poloxamer 188 have previously been assessed in order to alter their phase transition tendencies [13,22]. Moreover, it has been noted that combining PX with polymeric additives such as poly(acrylic acid) and poly(acrylic acid) derivatives might alter the T_gel_ of a gelling vehicle based on PX [23,24,25]. Early studies were particularly concerned with the impact of polymeric additives on the self-assembly and gelation of PX. In addition to synthetic polymers, proteins can interact with amphiphilic copolymers, altering their self-assembly properties [26].

Bovine serum albumin (BSA) is a hydrophilic globular protein utilized as a nutrient in cell culture. It has a wide range of biochemical and pharmaceutical applications because it is biocompatible, affordable, and readily available [27,28,29]. It has been reported that the presence of albumin that simulated the abundant protein in inflamed synovia influenced the phase transition of PX [30]. Furthermore, Neacsu et al. discovered that human serum albumin (HSA), which has a high sequence homology as well as a high 3D structure similarity to BSA [31], affected the liquid-to-gel transition of PX [32]. Recently, the opportunities of developing thermoresponsive formulations for the delivery of BSA-based therapeutics have been explored and suggested for use as an injectable platform [33]. Understanding the influence of additives on the self-assembly of PX is necessary for the rationale design of prospective thermoresponsive gels. More information is required to comprehend how BSA affects the micellization and gelation of PX.

In this study, the impact of BSA on the micellar properties and gelation of PX was examined. Using isothermal titration calorimetry (ITC), the aqueous associated colloids from PX and the mixtures were characterized, and the CMC was determined. Further, response surface methodology was applied to rationally design the experiments and statistically examine the relationship between the composition and the thermoresponsive micellization and gelation characteristics. To comprehend the phenomena of temperature-induced micellization and thermogelation of PX in the presence of BSA, differential scanning calorimetry (DSC) and rheological investigations were conducted.

## 2. Materials and Methods

### 2.1. Materials and Sample Preparation

PX was purchased from Sigma-Aldrich (Saint Louis, MO, USA). BSA was supplied by Fisher BioReagents (Thermo Fisher Scientific, Waltham, MA, USA). Ultrapure water was obtained from a Simplicity^®^ water purification system (Millipore, Molsheim, France) and used as the solvent for all samples.

The plain PX solutions were prepared using the cold method [34], which involved dispersing the desired quantity of PX in two-thirds of the required cold water while stirring to obtain a homogenous dispersion. The sample’s volume was then adjusted to the desired total volume by adding the remaining cold water. To prepare the mixtures of BSA and PX, the required quantity of BSA was dissolved in the PX solution before adjusting the volume of each mixture to the final volume. In order to prepare BSA solutions, the necessary amount of BSA was dissolved in water.

### 2.2. ITC Experiments

In order to ensure that the polymer concentration in the sample cell varies to span the micellization process as the titration progresses, the concentration of the amphiphilic polymer placed into the injection syringe as titrant should be higher than the CMC [35]. In addition, the heat change in each titration should not exceed the measuring capacity or be too small compared to background noise [35]. According to our preliminary study, 1.5% *w*/*v* PX was suitable for the ITC experiment. For studying the micellization of plain PX, the sample cell was initially loaded with water while 1.5% *w*/*v* PX was loaded into the syringe. Then the PX solution was successively titrated into water.

To examine the impact of BSA on PX micellization, the mixture of PX and BSA was continuously titrated into water that contained the same BSA composition as in the titrant. In this manner, the concentration of BSA remains constant during the titration procedure, and any heat change resulting from BSA dilution can be ruled out [6]. The mixtures of PX and BSA used as titrants were 0.75% and 1.5% *w*/*v* of BSA in 1.5% *w*/*v* PX.

The ITC experiments were conducted at 30 °C using a MicroCal PEAQ-ITC microcalorimeter (Malvern Panalytical, Malvern, UK). An initial small injection of 0.4 μL was followed by 38 injections of 1 μL of the titrant. Spacing was 120 s, and stirring speed was set to 750 rpm. All experiments were performed in triplicate. Data were processed and analyzed using MicroCal PEAQ-ITC Analysis Software and MATLAB 2018a (Mathworks, Natick, MA, USA). The CMC value was estimated from the maximum of the first derivative of the calorimetric titration curve, as described previously [35,36].

### 2.3. Response Surface Methodology

Response surface methodology with a 3-level factorial design was adopted to study how the factors affect responses including the CMT, the T_gel_, and the elastic modulus at 37 °C (G′_37°C_), which reflected gel strength [15]. The variables used for 3-level factorial design are represented in Table 1. The factors were the concentrations of PX and BSA in the thermoresponsive formulations. The actual values for low, medium, and high levels of PX were 16, 18, and 20% *w*/*v*, respectively. The concentration of 16% *w*/*v* was chosen as the low level since it is fractionally greater than the minimum concentration that forms gel as reported in the literature [12]. According to Table 1, the chosen highest concentration of BSA was 1.5% *w*/*v* which covered the BSA concentration generally used for biomedical and pharmaceutical applications [14,37,38]. The sample notation and description are listed in Table 2. With Design-Expert^®^ software (version 13; Stat-Ease Inc., Minneapolis, MN, USA), thirteen runs for three levels, two factors, and five center points were produced as shown in Table 3. The relations between the factors and the experimental results of the responses were established. Analysis of variance (ANOVA) was used to figure out whether the regression model was statistically significant. Statistical significance was regarded as a *p*-value of less than 0.05.

### 2.4. DSC Experiments

The DSC investigations were conducted using a Mettler Toledo DSC 3+ model (Mettler-Toledo, Viroflay, France). Samples were placed into aluminum pans with a pinhole in the lid. Temperature scans from 5 to 45 °C under nitrogen purge were conducted at a heating rate of 1 °C/min. STARe Evaluation Software was used for data analysis. All measurements were in triplicate.

### 2.5. Rheological Experiments

A HAAKE MARS 40 rheometer (ThermoFisher Scientific, Bremen, Germany) was used to evaluate the rheological characteristics. By utilizing a 60 mm parallel plate geometry with a 0.5 mm gap, the dynamic moduli—the elastic modulus (G′) and viscous modulus (G″)—were evaluated through temperature ramps at a heating rate of 1 °C/min from 5 to 45 °C. At a frequency of 1 Hz, the oscillation temperature ramp tests were conducted within the linear viscoelastic region. All measurements were in triplicate.

### 2.6. Statistical Analysis

ANOVA together with *post hoc* test was used to assess whether there were significant differences between the groups. A statistical difference was considered significant for *p*-value < 0.05.

## 3. Results and Discussion

### 3.1. ITC Measurements on the Self-Organization of PX into Micelles

ITC analysis is a precise method for monitoring the self-organization of the amphiphilic molecules and for determining CMC [35,39]. This method enables the observation of micellization in real time without the addition of dye molecules or measurements of surface tension [39]. Small amounts of a concentrated polymeric surfactant solution are titrated into the sample cell containing dispersion medium. This causes demicellization with every injection until the polymer concentration reaches CMC, which can be evaluated from the calorimetric titration data.

#### 3.1.1. PX Self-Organization in Water

**Figure 1:**
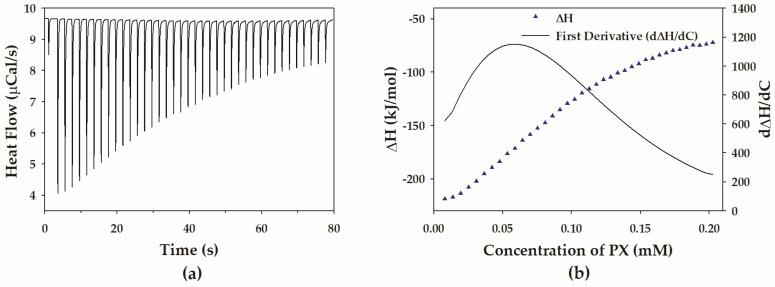
(**a**) Raw ITC thermogram of 1.5% *w*/*v* PX titrated into water at 30 °C. (**b**) Normalized titration curve of 1.5% *w*/*v* PX titrated into water at 30 °C. The first derivative of the titration curve is represented by a solid line.

Figure 1a represents the raw ITC thermogram of aqueous micellar PX solution titrated into water. Each injection caused a negative heat flow that deviated from the baseline, suggesting that the injection of micellar PX solution into water produced the exothermic heat flow effect. The concentration of the PX solution in the syringe was much higher than the expected CMC of PX; therefore, it may be inferred that the polymer solution in the syringe consisted of micellar colloids due to the self-organization. After the first injection of micellar solution from the syringe into the dispersion medium, the concentration of PX in the sample cell was far below CMC owing to the dilution of the injected PX solution. The dilution of the injected PX solution inside the sample cell destabilized the micellar colloids and triggered the micelle dissociation into unimers. This particular phenomenon is described as demicellization [35]. Therefore, the first few injections resulted in a large heat release (Figure 1a), which was attributed to the micelle dilution, the dissociation of the injected micelles into unimers, and the unimer dilution [35]. With subsequent injections, the magnitude of the observed heat release decreased. Eventually, the heat flow became almost constant for the last few injections.

The normalized titration curve was obtained by integrating the heat effect over time and subsequent normalization per amount of polymer. According to Figure 1b, the normalized titration curve of aqueous micellar PX solution titrated into water has a sigmoidal shape, which can be divided into three regions: a pre-micellar region, a transition concentration region, and a post-micellar region [35,39,40]. In the pre-micellar region, the concentration of PX in the sample cell was much below the CMC, and the observed enthalpy change was associated with the breakup of the added micelles as well as the dilution of unimers. As more of the concentrated PX was injected, the concentration of PX in the sample cell increased to the transition concentration region. The majority of injected micelles remained in the micellar form, with only a small portion dissociating into unimers in this region. The micelles gradually depress dissociating in this concentration range, as evidenced by the progressive decline in the enthalpy change magnitude. The PX concentration in the sample cell reaches the CMC in the transition concentration region. The differential curve (Figure 1b) was obtained by calculating the first derivative of the titration profile, and the CMC value was taken to be the concentration at which the differential curve reached its maximum. The CMC value of PX determined in this study was 0.057 ± 0.003 mM (0.072 ± 0.004 % *w*/*v*) which is in line with the previously reported range of CMC values [10,11,41]. In the post-micellar area, a plateau was reached on the titration curve. The predominant process occurring in the post-micellar region is the dilution of the PX micelles when the micelles no longer dissociate into unimers. The small heat release in this region reflected the dilution enthalpy of micelles.

#### 3.1.2. Effect of BSA on PX Self-Organization

**Figure 2:**
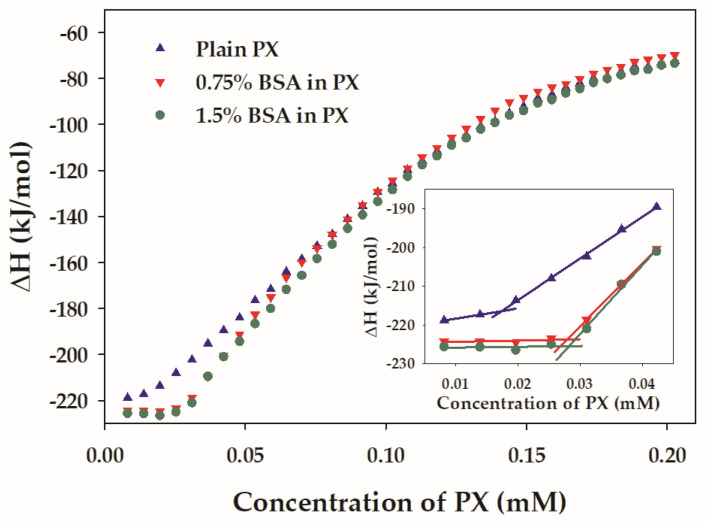
Normalized titration curve of 1.5% *w*/*v* PX (plain PX) titrated into water and the mixtures of 1.5% *w*/*v* PX and BSA titrated into water with the same BSA content at 30 °C. The BSA concentration for each mixture is shown in the legend.

The normalized titration curves of PX in the absence and presence of BSA are shown in Figure 2. The titration curves of PX in the presence of BSA exhibited a negative enthalpy change, indicating that the demicellization of PX in the presence of BSA remained the exothermic attribute. While having the sigmoidal shape, the concentration range of the pre-micellar region of PX in the presence of BSA was greater than that of plain PX. Using the concentration at the intersection of the extrapolated initial and linear ascent lines (inset of Figure 2) [42], the end of the pre-micellar region was estimated. The concentrations at the end of the pre-micellar region for plain PX, PX containing 0.75% *w*/*v* BSA, and PX containing 1.5% *w*/*v* BSA were 0.017 ± 0.001, 0.028 ± 0.000, and 0.026 ± 0.002 mM, respectively. Both samples containing BSA had significantly higher concentrations at the end of the pre-micellar region than plain PX (*p* < 0.05). The CMC of PX containing 0.75% *w*/*v* BSA, and PX containing 1.5% *w*/*v* BSA were 0.054 ± 0.003 and 0.052 ± 0.003 mM, respectively. For the CMC values, there was no statistical difference among plain PX, PX containing 0.75% *w*/*v* BSA, and PX containing 1.5% *w*/*v* BSA. The interaction between BSA and PX unimers may play a role in the shift of the transition from the pre-micellar region to the transition concentration region. It has been suggested that the hydrophobic interaction between PPO residues of PX unimers and BSA induced the PPO globule of PEO-PPO-PEO block copolymers to expand in water [43]. The corresponding interaction might retard the association between PPO blocks at low concentrations of PX. Therefore, the presence of BSA extended the concentration range of the pre-micellar region.

### 3.2. Investigation on the Temperature-Induced Micellization and Gelation

The response surface methodology based on the three-level factorial design is a practical approach that uses a reasonable number of experiments and statistical analysis to establish a model correlating the casual factors with studied responses [44]. The potential effect of BSA on the temperature-induced micellization and gelation of PX was explored using the response surface approach. The DSC experiments were performed to monitor the temperature-induced micellization, and the thermoresponsive viscoelasticity of the samples was evaluated to describe the temperature-induced gelation.

The three-level factorial design was implemented to statistically identify and quantify the impact of two factors, the concentrations of PX (X_1_) and BSA (X_2_), on the responses including the CMT (Y_1_), the T_gel_ (Y_2_) and the G′_37°C_ (Y_3_). The three-level factorial design and response data are shown in Table 3. The sample notation is also listed in Table 3.

#### 3.2.1. DSC Thermograms and Temperature Ramp Rheograms

**Figure 3:**
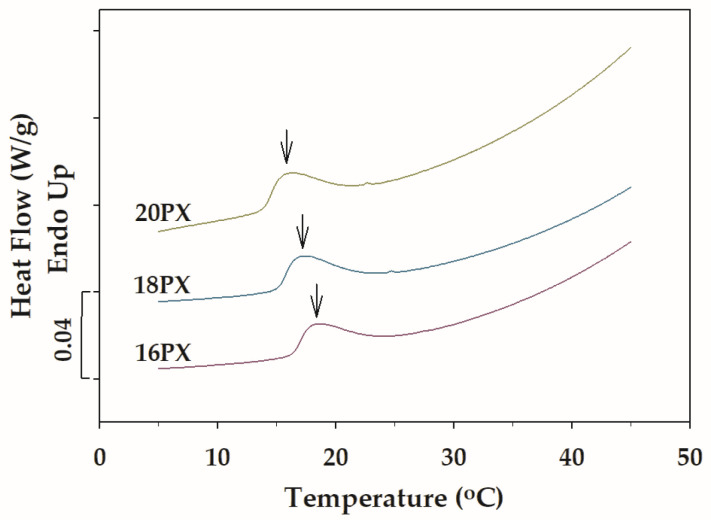
DSC thermograms of aqueous PX at various concentrations (16PX, 18PX, and 20PX). Arrows denote the critical micellization temperature.

**Figure 4:**
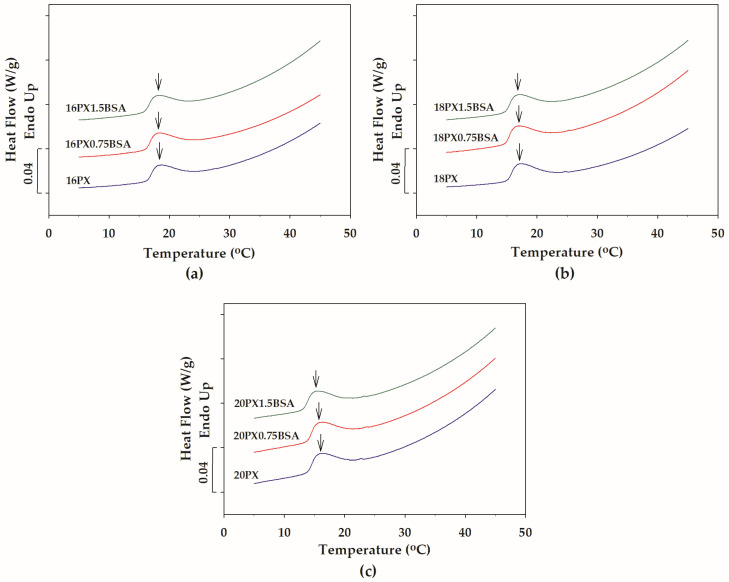
DSC thermograms of (**a**) 16PX and mixtures containing 16PX and various concentrations of BSA (16PX0.75BSA and 16PX1.5BSA), (**b**) 18PX and mixtures containing 18PX and various concentrations of BSA (18PX0.75BSA and 18PX1.5BSA), and (**c**) 20PX and mixtures containing 20PX and various concentrations of BSA (20PX0.75BSA and 20PX1.5BSA). Arrows denote the critical micellization temperature.

The temperature-dependent micellization of PX in the presence and absence of BSA was investigated using DSC. The DSC thermograms for plain PX samples—16PX, 18PX, and 20PX—are depicted in Figure 3. The temperature-induced dehydration of PX molecules led to the association of the hydrophobic PPO blocks, resulting in the formation of PX micelles composed of PPO cores and PEO coronas [5,13]. A broad endotherm observed in the DSC thermogram of PX could be attributed to the micellization [13,22]. In response to heating, the onset of the endothermic signal corresponded to the initiation of micelle formation, and the end of the endotherm reflected the completion of the micellization process. In general, the CMT is defined as the temperature at which the endothermic signal reaches its peak [5,45]. Evidence for this temperature-induced micellization was provided by the endothermic traces for all plain PX as shown in Figure 3. The CMT as indicated by arrows in Figure 3 was slightly shifted to lower temperature with increasing PX concentration, indicating the concentration-facilitated self-association of amphiphilic co-polymers as previously described [46,47]. Figure 4 displays the DSC traces for the temperature-induced micellization of PX in the presence of BSA. The thermal characteristic of PX micellization was not noticeably altered by the addition of BSA.

**Figure 5:**
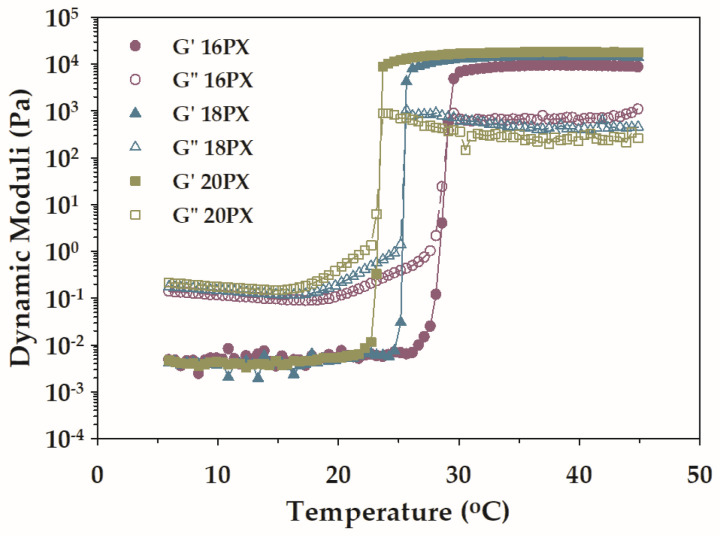
Temperature-dependent dynamic moduli of aqueous PX at various concentrations (16PX, 18PX, and 20PX).

**Figure 6:**
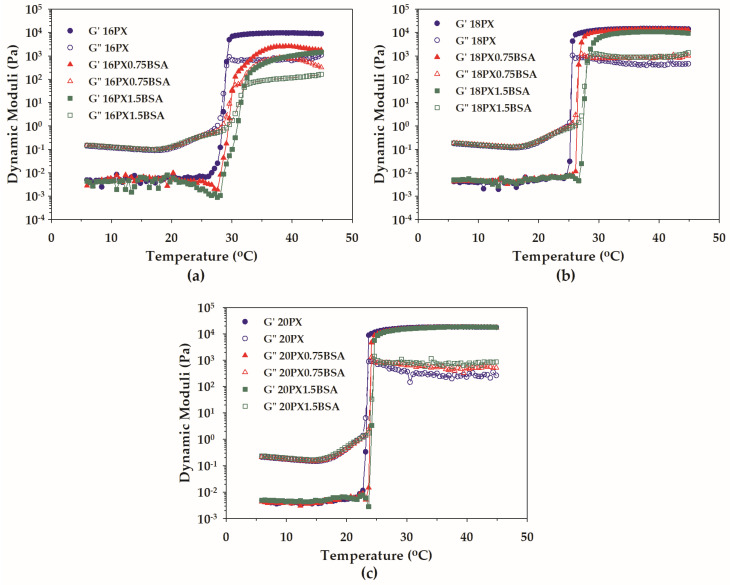
Temperature-dependent dynamic moduli of (**a**) 16PX and mixtures containing 16PX and various concentrations of BSA (16PX0.75BSA and 16PX1.5BSA), (**b**) 18PX and mixtures containing 18PX and various concentrations of BSA (18PX0.75BSA and 18PX1.5BSA), and (**c**) 20PX and mixtures containing 20PX and various concentrations of BSA (20PX0.75BSA and 20PX1.5BSA).

Temperature ramp rheometry was performed to examine how BSA affected the thermoresponsive gel formation of PX. The variation of the dynamic moduli with temperature for plain PX and the mixtures of PX and BSA is depicted in Figure 5 and Figure 6. All samples exhibited thermogelation behavior. At low temperatures, the values of G′ were below those of G″ for all plain PX (Figure 5). This reflected the liquid stage of the sample. Both dynamic moduli increased with increasing temperature, and the values of G′ became superior to those of G″ at high temperatures. The predominant G′ at high temperatures revealed the gel characteristic of all samples. The liquid-to-gel transition took place in the temperature range at which the abrupt increase in G′ was detected, and the temperature at which the crossover of G′ and G″ was observed was denoted as the T_gel_ [13,22]. From Figure 5, increasing PX concentration caused the crossover points of G′ and G″ to shift to a lower temperature. This indicated that the thermoresponsive gelation was facilitated by raising the concentration of PX. On the contrary, the presence of BSA resulted in a shift of the crossover points of G′ and G″ to a higher temperature (Figure 6). The gelation of PX could be attributed to the inter-micellar entanglements caused by raising the temperature [48]. The high concentration of PX produced a large number of micelles available for the formation of the gel structure; therefore, the increase in the PX concentration facilitated the temperature-induced gelation. The increase in gelation temperature with the incorporation of BSA could be associated with the steric hindrance of serum albumin. The relationship between the micellization and gelation characteristics, and the concentration of each component was further determined using response surface methodology based on the three-level factorial design.

#### 3.2.2. Experimental Data and Response Surface Modeling of Three-Level Factorial Design

The experimental data presented in Table 3 were fitted to linear, two-factor interaction (2FI), and quadratic models to obtain the regression equation. For checking model adequacy, the comparative values of sequential *p*-value, lack of fit *p*-value, correlation coefficient (*R*^2^), adjusted *R*^2^, and predicted *R*^2^ are summarized in Table 4. When the sequential *p*-value is lower than 0.05 and a non-significant lack of fit (*p*-value > 0.05) is obtained, the model is considered to be adequate [49]. In addition, the feasibility of the model is determined by the values of *R*^2^, adjusted *R*^2^, and predicted *R*^2^. The adjusted *R*^2^ and the predicted *R*^2^ should be close to the same or different by no more than 0.2 [50,51]. All *R*^2^ values of the satisfactory model should fall within the range of 0.7 to 1.0, and the adjusted *R*^2^ should be close to 1.0 [50,51]. Accordingly, the response Y_1_ was found to follow the linear model, while the quadratic models were selected to describe the effects of the factors on the responses Y_2_ and Y_3_.

The relationship between the CMT and the concentrations of PX and BSA was represented by the following linear model in terms of coded factors.

**Figure 7:**
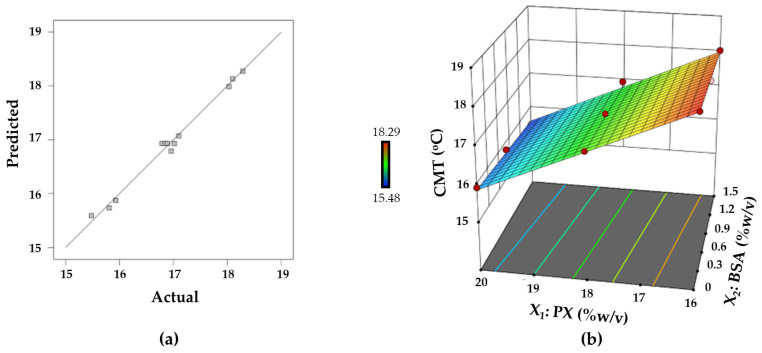
(**a**) Linear plot between predicted and actual response values for the critical micellization temperature, and (**b**) response surface plot representing the effect of PX and BSA on the critical micellization temperature.

Y_1_ = **16.93** − **1.20X_1_** − 0.1417X_2_ (1)

whereas a negative sign of a factor denotes an inverse relationship between the factor and the response, a positive sign of a factor signifies a positive effect of the factor on the response. In an equation, any term with statistical significance is bolded. As illustrated in Figure 7a, the plot of experimental response against predicted response shows an adequate correlation and behaves with a uniform distribution of data points around the 45° line. A high correlation between predicted and actual experimental data indicated that the model was reliable [52]. According to the response surface plot in Figure 7b, the CMT was mainly dependent on the concentration of PX. As the concentration of PX increased, the CMT decreased in a linear fashion. Although the concentration of BSA also had a negative effect on the CMT, the effect of BSA concentration on the response Y_1_ was minor and insignificant. The insignificant influence of BSA on the CMT found in this study is in accordance with a study by Perinelli et al., which investigated the effect of BSA on the CMT of PX at low PX concentrations (2.5% and 5% *w*/*w*) in phosphate buffer and found no notable effect on the CMT value of PX [30].

The relationship between the T_gel_ and the concentrations of PX and BSA was represented by the following quadratic model in terms of coded factors.

Y_2_ = **26.81 − 3.24X_1_ + 1.06X_2_ − 0.4450X_1_X_2_ + 0.4290X_1_^2^** − 0.0810X_2_^2^ (2)

**Figure 8:**
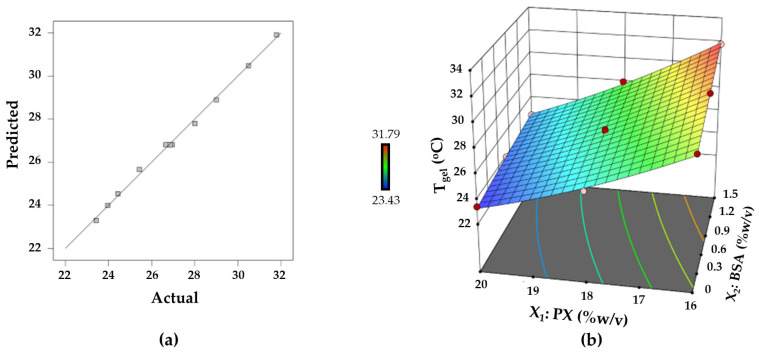
(**a**) Linear plot between predicted and actual response values for the gelation temperature, and (**b**) response surface plot representing the effect of PX and BSA on the gelation temperature.

The coefficient of a linear term (X_1_ or X_2_) represents the effect of a particular factor, whereas the coefficients of an interactive term (X_1_X_2_) and a quadratic term (X_1_^2^ or X_2_^2^) describe the interaction between two factors and quadratic effect, respectively. In Equation (2) X_1_, X_2_, X_1_X_2_, and X_1_^2^ were significant model terms, as highlighted in bold. When the experimental response is plotted against the predicted response (Figure 8a), the data points are close to the straight line. This reflected an adequate agreement between the experimental data and the predicted responses. According to Equation (2), both PX and BSA concentrations significantly impacted on the T_gel_. The increase in the PX concentration lowered T_gel_, as revealed by the negative coefficient of its linear term. This is consistent with the gelling behavior of PX reported by Liu et al. [48]. With the increase in PX concentration, the polymer solution gains in density and volume fraction of micelles. As a result, the distance between micelles was reduced, favoring the temperature-induced formation of a micellar network [48]. Hence, the increase in PX concentration gave rise to the reduction of T_gel_. The positive coefficient for the linear term of BSA, on the other hand, showed that increasing the concentration of BSA shifted T_gel_ to higher values. As a result, these two factors have opposing effects. However, the curvature depicted on the response surface plot (Figure 8b) indicates the complexity of the interaction between the components in the mixtures. The influence of one component may vary depending on the amount of another in the mixture. At the lower level of PX, BSA had a more noticeable effect on T_gel_ increase. The steric hindrance caused by the additives may be responsible for the increase in T_gel_ of the PX-based mixtures [15,30]. The presence of BSA could produce steric hindrance and hinder the temperature-induced organization of PX micelles into the inter-micellar packing, causing a positive effect on T_gel_. As discussed previously, the density and volume fraction of PX micelles depended on the concentration of PX. The micellar volume fraction of PX at low concentration may be low compared with that of PX at high concentration. Therefore, the addition of BSA to PX at a low concentration had a more pronounced effect on T_gel_.

The relationship between the G′_37°C_ and the concentrations of PX and BSA was represented by the following quadratic model in terms of coded factors.

Y_3_ = **12602.36 + 7238.56X_1_ − 2177.17X_2_ + 2015.76X_1_X_2_ − 1922.08X_1_^2^** + 1070.08X_2_^2^ (3)

**Figure 9:**
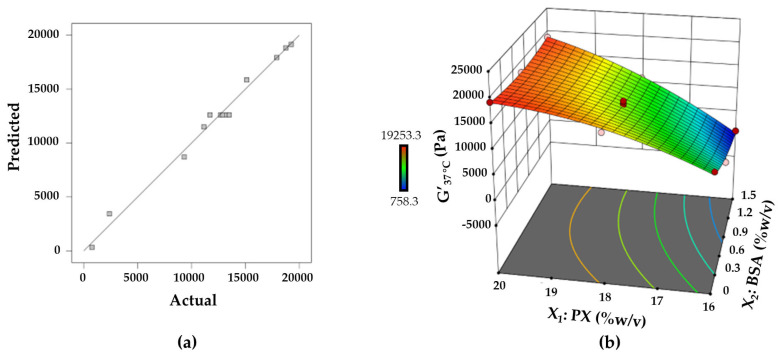
(**a**) Linear plot between predicted and actual response values for the elastic modulus at 37 °C, and (**b**) response surface plot representing the effect of PX and BSA on the elastic modulus at 37 °C.

X_1_, X_2_, X_1_X_2_, and X_1_^2^ were significant terms for the storage modulus at 37 °C. Negative coefficients of X_2_ and X_1_^2^ indicated the inverse relationship for the G′_37°C_ while other terms showed a positive effect on the G′_37°C_. The adequate agreement between the experimental data and the predicted responses is demonstrated in Figure 9a. The response surface plot is depicted in Figure 9b to further highlight the relationship between these factors and the response. As can be seen, increasing the concentration of PX led to a significant increase in the G′_37°C_. For PX-based gels, the increment of the elastic characteristic is associated with the increase in inter-micellar entanglements, which promote the deformation resistance of the gels [53]. The increase in PX concentration produced more entanglements in the gel structure, enhancing the gel strength. The surface response of the G′_37°C_ tended to tilt down with increasing BSA concentration. In addition, the complexity of the interplay between the components was shown by the curvature of the response surface plot. At the lower level of PX, the effect of BSA on the G′_37°C_ is more prominent. The reduction in elasticity of gels has been reported for the mixtures of PX and poly(acrylic acid) and the mixtures of PX and HSA [23,32]. This softening effect could be explained by the attachment of the macromolecules to the micelles, which in turn limited the movement of the micelles and obstructed inter-micellar entanglements.

## 4. Conclusions

The micellization of PX in the presence and absence of BSA was investigated using ITC. The pre-micellar region, the transition concentration region, and the post-micellar region were observed in the calorimetric titration curves. The presence of BSA did not show a discernible effect on the CMC of PX. However, the addition of BSA caused the extension of the pre-micellar region. This could be attributed to the hydrophobic interaction between PPO residues of PX unimers and BSA. In addition to the self-organization of PX at a certain temperature, the temperature-dependent micellization and gelation of PX were also investigated. The presence of BSA did not show a significant effect on the CMT but altered the T_gel_ as well as the gel integrity of PX-based solutions. Response surface methodology represented the linear relationship between the composition and the CMT. The change in the CMT of the mixtures was mainly attributed to the concentration of PX. The complex interplay between PX and BSA was found for the T_gel_ and the G′_37°C_. BSA interfered in the inter-micellar entanglements into gel structure. Accordingly, the presence of BSA showed the modulation effect on the T_gel_ and the softening effect on the gel integrity. Understanding the impact of serum albumin on the self-assembly and gelling characteristics of PX would allow the development of thermoresponsive systems with controlled gelation temperatures and viscoelasticity for drug delivery and tissue engineering.

## Figures and Tables

**Table 1:** Variables used for 3-level factorial design.

Factors	Actual Level (Coded Level)		
	Low	Medium	High
X_1_: PX concentration (% *w*/*v*)	16 (−1)	18 (0)	20 (+1)
X_2_: BSA concentration (% *w*/*v*)	0 (−1)	0.75 (0)	1.5 (+1)
**Responses**			
Y_1_: CMT (°C)			
Y_2_: T_gel_ (°C)			
Y_3_: G′_37°C_ (Pa)			

**Table 2:** List of sample notation and description.

Sample Notation	Description of Sample
16PX	16% *w*/*v* PX
16PX0.75BSA	The mixture containing 16% *w*/*v* PX and 0.75% *w*/*v* BSA
16PX1.5BSA	The mixture containing 16% *w*/*v* PX and 1.5% *w*/*v* BSA
18PX	18% *w*/*v* PX
18PX0.75BSA	The mixture containing 18% *w*/*v* PX and 0.75% *w*/*v* BSA
18PX1.5BSA	The mixture containing 18% *w*/*v* PX and 1.5% *w*/*v* BSA
20PX	20% *w*/*v* PX
20PX0.75BSA	The mixture containing 20% *w*/*v* PX and 0.75% *w*/*v* BSA
20PX1.5BSA	The mixture containing 20% *w*/*v* PX and 1.5% *w*/*v* BSA

**Table 3:** The 3-level factorial design and response data.

Run	Sample Notation	Factors		Responses *		
		X_1_	X_2_	Y_1_	Y_2_	Y_3_
1	16PX0.75BSA	16	0.75	18.10	30.50	2375.67
2	18PX0.75BSA	18	0.75	16.85	26.66	13,493.30
3	20PX	20	0	15.93	23.43	19,253.30
4	18PX0.75BSA	18	0.75	16.86	26.96	12,910.00
5	18PX0.75BSA	18	0.75	16.89	26.87	11,713.30
6	18PX1.5BSA	18	1.5	16.96	28.01	11,140.00
7	20PX0.75BSA	20	0.75	15.81	23.96	17,900.00
8	16PX1.5BSA	16	1.5	18.03	31.79	758.30
9	18PX0.75BSA	18	0.75	16.79	26.76	13,286.70
10	16PX	16	0	18.29	29.00	9331.33
11	18PX0.75BSA	18	0.75	17.02	26.83	12,693.30
12	18PX	18	0	17.10	25.43	15,120.00
13	20PX1.5BSA	20	1.5	15.48	24.44	18,743.30

* Mean value of triplicate experiments.

**Table 4:** Statistic comparison of regression models for responses Y_1_, Y_2_, and Y_3_.

Models	Sequential *p*-Value	Lack of Fit *p*-Value	*R* ^2^	Adjusted *R*^2^	Predicted *R*^2^	Remarks
**Response Y_1_**						
Linear model	<0.0001	0.3571	0.9892	0.9871	0.9817	Suggested
2FI model	0.3574	0.3403	0.9903	0.9870	0.9793	
Quadratic model	0.5725	0.2507	0.9917	0.9858	0.9516	
**Response Y_2_**						
Linear model	<0.0001	0.0064	0.9786	0.9743	0.9483	
2FI model	0.0126	0.0197	0.9897	0.9862	0.9639	
Quadratic model	0.0119	0.1040	0.9971	0.9950	0.9766	Suggested
**Response Y_3_**						
Linear model	<0.0001	0.0207	0.9161	0.8994	0.8994	
2FI model	0.0125	0.0618	0.9596	0.9461	0.9461	
Quadratic model	0.0150	0.2819	0.9878	0.9791	0.9791	Suggested

## Data Availability

Not applicable.
